# In-Motion Forward–Forward Backtracking Fine Alignment Based on Displacement Observation for SINS/GNSS

**DOI:** 10.3390/s24247916

**Published:** 2024-12-11

**Authors:** Yongyun Zhu, Yaohui Zhu, Xinhua Wei, Bingbo Cui, Shede Liu

**Affiliations:** 1 School of Agricultural Engineering, Jiangsu University, Zhenjiang 212013, China; yongyunzhu@ujs.edu.cn (Y.Z.); yaohui_zhu@ujs.edu.cn (Y.Z.); wxh@ujs.edu.cn (X.W.); 2 School of Instrument Science & Engineering, Southeast University, Nanjing 210096, China; 230208374@seu.edu.cn

**Keywords:** fine alignment, SINS/GNSS, forward–forward backtracking, displacement observation

## Abstract

To solve the problem of slow convergence seen in the traditional fine alignment algorithm based on linear Kalman filtering, a forward–forward backtracking fine alignment algorithm for SINS is proposed after reanalyzing the fine alignment model in this paper. First, the forward–forward backtracking fine alignment model in initial navigation frame was derived. The displacement vector of the carrier in the initial navigation frame solved by GNSS positioning was utilized as the observation of the fine alignment model. Second, under the premise of storing only part of the navigation data, the initial alignment convergence speed was improved by backtracking and reusing the navigation data. The experimental results of the simulation and vehicle tests showed that each backtracking alignment can improve the accuracy of the fine alignment to the performance requirements of the initial alignment, which proved the effectiveness and feasibility of the backtracking fine alignment algorithm proposed in this paper.

## 1. Introduction

Unmanned Ground Vehicles (UGVs) are capable of avoiding obstacles and operating autonomously in complex and harsh environments. They are widely used in transportation, reconnaissance, search, inspection, and other missions, driving advancement in automation and robotics technology [1,2]. Positioning and attitude determination technology is one of the key technologies for the autonomous driving and operation of UGVs [3,4,5]. Real-time and accurate detection of the position and attitude in complex operating environments is fundamental and crucial for the precise operation and safe return of UGVs. Commonly used attitude–position detection methods include the Global Navigation Satellite System (GNSS) [6,7], computer vision navigation [8,9], and the Strapdown Inertial Navigation System (SINS) [10,11]. Traditional single navigation and positioning technologies cannot meet the demand for the accurate and reliable positioning of UGVs in complex environments. Integrated navigation technologies have developed rapidly in many fields, particularly in the field of autonomous driving, which have become one of the core technologies for improving positioning accuracy and reliability. The integration of various navigation and positioning technologies, such as GNSS, SINS, visual navigation, and LiDAR, enables high-precision and stable positioning in complex environments [12,13,14,15]. Whether in urban areas and underground environments where GNSS signals are obstructed, or in open environments like highways, the combination of these technologies significantly enhances the reliability and safety of autonomous driving systems. SINS provides a reliable navigation solution for UGVs because of their strong autonomy, high update frequency, and good dynamic performance [16,17], which further enhances their operational capability in complex environments. By utilizing SINS as the core, combined with a GNSS-based attitude measurement system, precise and reliable attitude measurements can be provided for the autonomous driving of UGVs.

The SINS can utilize the raw data from the Inertial Measurement Unit (IMU) to determine the variation in the motion state relative to the initial state of the UGV. The initial motion state of a UGV includes its initial position, initial velocity, and initial attitude. The initial velocity and initial position of the carrier can be easily obtained by other auxiliary sensors such as a GNSS. Therefore, the initial attitude obtained from the solution of the initial alignment process is crucial for the subsequent navigation and localization process of the SINS [18,19]. Convergence speed and alignment accuracy are two important parameters in measuring the performance of the initial alignment algorithm [20,21].

Since UGVs are usually in motion when performing their missions, designing in-motion alignment algorithms with high accuracy and fast convergence is the key to improving the maneuvering operations of UGVs. In recent years, many scholars around the world have carried out comprehensive research on in-motion alignment methods for SINS [22,23,24]. In their research on coarse alignment methods aided by a GNSS, Y. Chen et al. explored a novel, fast, indirect in-motion coarse alignment method based on an adaptive Student’s t-based Kalman filter to solve the problems of the heavy computing cost and filtering instability [25]. To suppress the alignment errors caused by the maneuvering of the vehicle and the accumulation of inertial sensor errors, X. Xu proposed an improved in-motion alignment method for a SINS/GPS by integrating sliding windows [26]. In their research on fine alignment methods aided by a GNSS, S. Cao et al. proposed an adaptive flight alignment method based on observability analysis for a SINS/GPS polarized integrated navigation system to improve the accuracy and robustness of the alignment process [27].

**Figure 1:**
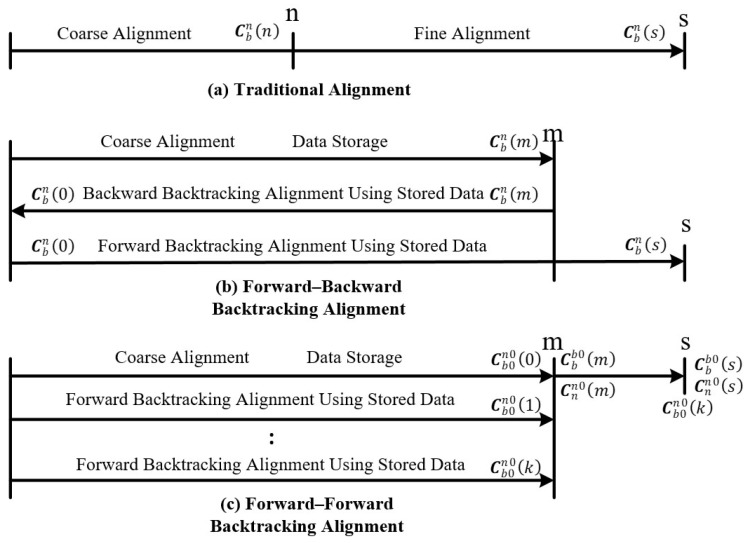
Schematic diagram of the principle of the backtracking alignment.

The traditional initial alignment process is usually divided into two stages chronologically, coarse alignment and fine alignment [28,29], as shown in Figure 1a. Each stage take place over a certain amount of time to ensure that the convergence speed and accuracy of the initial alignment algorithm meet the performance requirements. With the total initial alignment time fixed, if the time required for coarse alignment is reduced, the coarse alignment algorithm may not be able to converge to a smaller angular range, thus affecting the accuracy of fine alignment. Conversely, if the fine alignment time is reduced, while the performance of the coarse alignment can be maintained, it is difficult to achieve the desired accuracy of the fine alignment.

To improve the convergence speed and accuracy of fine alignment, a backtracking alignment strategy needs to be designed. The performance of the initial alignment can be improved by storing certain sensor data during the operation of the coarse alignment algorithm and reutilizing these raw data in the fine alignment stage [30]. With the continuous development and progress of computer technology, the storage capacity of computers has been greatly improved. The processing speed of computers has also been significantly improved, which provides a hardware foundation for the successful operation of the backtracking alignment strategy based on data storage.

Currently, there are two main types of backtracking alignment schemes based on data storage: forward–backward backtracking alignment and forward–forward backtracking alignment [31,32,33]. The structure of forward–backward backtracking fine alignment is shown in Figure 1b. L. Chang et al. [30] proposed a novel backtracking scheme that combines the recorded IMU raw data from the forward process and velocity information of GPS to construct vector observations for attitude-based initial alignment. This approach improved the accuracy and robustness of the initial alignment within a short period of time, and its effectiveness was validated through car-mounted experiments. To improve the initial alignment velocity of the SINS at the stationary base, Y. Lin et al. [31] proposed a forward–backward alignment method, which was verified by turntable tests. X. Wei et al. [34] proposed an improved alignment method based on backtracking navigation, which significantly improved the alignment performance of a low-cost SINS/GPS system by considering low-cost inertial sensor bias and correcting it with the cubic Kalman filter of the Lie Group. In order to overcome the limitation of focusing only on attitude alignment in traditional alignment methods, Y. Sun et al. [35] proposed a dynamic alignment method based on a backtracking scheme for a SINS/OD system, which achieved simultaneous high-precision acquisition of both attitude and position in a short period of time by combining the improved optimized coarse alignment and backtracking fine alignment. However, the forward–backward backtracking alignment algorithm needs to use all IMU raw data when performing backward backtracking fine alignment. Due to the high frequency of SINS updates, storing all the raw data during the alignment process consumes a large amount of storage space, resulting in a waste of resources and low computational efficiency.

For the forward backtracking fine alignment algorithm, if the traditional fine alignment error model under the navigation frame is used, it is also necessary to store all the IMU raw data, which will inevitably increase the consumption of computer storage space. Therefore, using the backtracking fine alignment method, a forward–forward backtracking fine alignment method based on an inertial-frame error model was designed in this paper. By analyzing the inertial-frame coarse alignment method, a new backtracking fine alignment error model was established, which enabled the fine alignment process to be completed quickly and accurately under the condition that only some of the data were stored.

The remainder of this paper is organized as follows. An overview of the model of backtracking fine alignment is given in Section 2. The effectiveness and practicability of the proposed algorithm is demonstrated through simulation and vehicle tests in Section 3 and Section 4. Finally, conclusions are drawn in Section 5.

## 2. The Error Model of Backtracking Fine Alignment Based on an Inertial Frame

In this section, the derivation of a forward–forward backtracking fine alignment error model based on an inertial frame is given. The body frame of the IMU is denoted as the b-frame. The geographic frame selected as the navigation frame is denoted as the n-frame. The b0-frame denotes the inertial non-rotating frame aligned with the b-frame at *t*0. The n0-frame denotes the inertial non-rotating frame aligned with the n-frame at *t*0.

### 2.1. State Equation of Backtracking Alignment

After coarse alignment, the directional cosine matrix can be expressed as

(1) $${\tilde{C}}_{b}^{n}=C_{n0}^{n}{\hat{C}}_{b0}^{n0}{\tilde{C}}_{b}^{b0}$$

where ${\tilde{C}}_{b}^{n}$ denotes the attitude matrix with errors from the b-system to the n-system. $C_{n0}^{n}$ denotes the attitude matrix from the n0-system to the n-system. ${\hat{C}}_{b0}^{n0}$ denotes the attitude matrix with errors from the b0-system to the n0-system. ${\tilde{C}}_{b}^{b0}$ denotes the attitude matrix with errors from the b-system to the b0-system.

Since the carrier velocity error has a negligible effect on the update of the attitude matrix $C_{n0}^{n}$, the attitude matrix $C_{n0}^{n}$ calculated via coarse alignment can be considered error-free. Since the gyroscope output contains measurement error, the directional cosine matrix differential equation for the matrix ${\tilde{C}}_{b}^{b0}$ with error is expressed as

(2) $${\dot{\tilde{C}}}_{b\left(t\right)}^{b0}={\tilde{C}}_{b\left(t\right)}^{b0}\left[{\tilde{\omega }}_{ib}^{b}\;\times \right]$$

where ${\tilde{\omega }}_{ib}^{b}$ denotes the angular velocity vector with error measured using a gyroscope. The matrix ${\tilde{C}}_{b\left(t\right)}^{b0}$ can be expressed as the multiplication of the truth value matrix $C_{b\left(t\right)}^{b0}$ and the attitude error matrix ${\tilde{\phi }}^{b0}$.

(3) $${\tilde{C}}_{b\left(t\right)}^{b0}=\left[I_{3}-\left({\tilde{\phi }}^{b0}\times \right)\right]C_{b\left(t\right)}^{b0}$$

where ${\tilde{\phi }}^{b0}$ denotes the misalignment angle between the matrices ${\tilde{C}}_{b\left(t\right)}^{b0}$ and $C_{b\left(t\right)}^{b0}$.

The matrix ${\hat{C}}_{b0}^{n0}$ with error can be expressed as

(4) $${\hat{C}}_{b0}^{n0}=\left(I-{\hat{\phi }}^{n0}\times \right)C_{b0}^{n0}$$

where ${\hat{\phi }}^{n0}$ denotes the residual error of the coarse alignment algorithm. Equation (4) can be converted into the following form:

(5) $${\hat{C}}_{b0}^{n0}=\left(I-{\hat{\phi }}^{n0}\times \right)C_{b0}^{n0}=C_{b0}^{n0}C_{n0}^{b0}\left(I-{\hat{\phi }}^{n0}\times \right)C_{b0}^{n0}=C_{b0}^{n0}\left(I-{\hat{\phi }}^{b0}\times \right)$$

Substituting Equations (3) and (5) into Equation (1), collapsing and neglecting the second-order minors yields

(6) $$\begin{array}{cc} {\tilde{C}}_{b}^{n} & =C_{n0}^{n}C_{b0}^{n0}\left(I-{\hat{\phi }}^{b0}\times \right)\left(I-{\tilde{\phi }}^{b0}\times \right)C_{b}^{b0}\approx C_{n0}^{n}C_{b0}^{n0}\left(I-\left({\hat{\phi }}^{b0}+{\tilde{\phi }}^{b0}\right)\times \right)C_{b}^{b0}\\ & =C_{n0}^{n}C_{b0}^{n0}\left(I-\left({\hat{\phi }}^{b0}+{\tilde{\phi }}^{b0}\right)\times \right)C_{n0}^{b0}C_{b0}^{n0}C_{b}^{b0}=C_{n0}^{n}\left(I-\left({\hat{\phi }}^{n0}+{\tilde{\phi }}^{n0}\right)\times \right)C_{b0}^{n0}C_{b}^{b0} \end{array}$$

The attitude error $\phi^{i}$ is denoted as the following:

(7) $$\phi^{i}={\hat{\phi }}^{n0}+{\tilde{\phi }}^{n0}$$

Then, Equation (6) can be re-expressed as

(8) $${\tilde{C}}_{b}^{n}\approx C_{n0}^{n}\left(I-\phi^{i}\times \right)C_{b0}^{n0}C_{b}^{b0}$$

The derivate of Equation (7) is expressed as

(9) $${\dot{\phi }}^{i}={\dot{\hat{\phi }}}^{n0}+{\dot{\tilde{\phi }}}^{n0}$$

where ${\hat{\phi }}^{n0}$ is the constant-value attitude error. Thus,

(10) $${\dot{\hat{\phi }}}^{n0}=0$$

The derivate of attitude error ${\tilde{\phi }}^{n0}$ is expressed as

(11) $${\dot{\tilde{\phi }}}^{n0}=-C_{b0}^{n0}C_{b}^{b0}\left(\varepsilon^{b}+w_{g}^{b}\right)$$

Combining Equations (9)–(11), the attitude error equation for backtracking fine alignment can be derived as

(12) $${\dot{\phi }}^{i}={\dot{\hat{\phi }}}^{n0}+{\dot{\tilde{\phi }}}^{n0}=-C_{b0}^{n0}C_{b}^{b0}\left(\varepsilon^{b}+w_{g}^{b}\right)$$

where $\varepsilon^{b}$ represents the constant bias of gyroscopes and $w_{g}^{b}$ represents the random noise of gyroscope measurement.

From the specific force equation, an equation can be deduced as the following:

(13) $${\dot{V}}^{n0}=C_{b0}^{n0}C_{b}^{b0}f^{b}-\left(2\omega_{ie}^{n0}+\omega_{en0}^{n0}\right)\times V^{n0}+C_{n}^{n0}g^{n}$$

where $V^{n0}$ denotes the projection of the carrier velocity in the n0-frame. ${\dot{V}}^{n0}$ denotes the differential form of the velocity vector. $f^{b}$ denotes the specific force measured using an accelerometer. $\omega_{ie}^{n0}$ denotes the projection of the rotational angular velocity of the Earth frame with respect to the inertial frame under the n0-frame. $\omega_{en0}^{n0}$ denotes the projection of the rotational angular velocity of the initial frame with respect to the Earth frame under the n0-frame. $g^{n}$ denotes the gravity accelerometer vector.

(14) $$\omega_{ie}^{n0}+\omega_{en0}^{n0}=\omega_{in0}^{n0}=0$$

Thus, Equation (13) can be re-expressed as

(15) $${\dot{V}}^{n0}=C_{b0}^{n0}C_{b}^{b0}f^{b}-\omega_{ie}^{n0}\times V^{n0}+C_{n}^{n0}g^{n}$$

In practical application environments, the specific force equation with errors is expressed as

(16) $${\dot{\tilde{V}}}^{n0}={\hat{C}}_{b0}^{n0}{\tilde{C}}_{b}^{b0}{\tilde{f}}^{b}-{\tilde{\omega }}_{ie}^{n0}\times {\tilde{V}}^{n0}+C_{n}^{n0}g^{n}$$

In the above equation, the attitude matrix $C_{n}^{n0}$ and gravity vector $g^{n}$ remain unchanged due to the small change in the position of the UGV in a short period of time.

Subtracting Equation (16) from Equation (15) yields

(17) $$\begin{array}{cc} \delta {\dot{V}}^{n0} & ={\dot{\tilde{V}}}^{n0}-{\dot{V}}^{n0}={\hat{C}}_{b0}^{n0}{\tilde{C}}_{b}^{b0}{\tilde{f}}^{b}-{\tilde{\omega }}_{ie}^{n0}\times {\tilde{V}}^{n0}+C_{n}^{n0}g^{n}-\left(C_{b0}^{n0}C_{b}^{b0}f^{b}-\omega_{ie}^{n0}\times V^{n0}+C_{n}^{n0}g^{n}\right)\\ & =C_{b0}^{n0}\left(I-{\hat{\phi }}^{b0}\times \right)\left(I-{\tilde{\phi }}^{b0}\times \right)C_{b}^{b0}\left(f^{b}+\nabla^{b}+w_{a}^{b}\right)\\ & -C_{b0}^{n0}C_{b}^{b0}f^{b}-\left[\left(\omega_{ie}^{n0}+\delta \omega_{ie}^{n0}\right)\times \left(V^{n0}+\delta V^{n0}\right)-\omega_{ie}^{n0}\times V^{n0}\right]\\ & \approx \left(I-\phi^{i}\times \right)C_{b0}^{n0}C_{b}^{b0}\left(f^{b}+\nabla^{b}+w_{a}^{b}\right)-C_{b0}^{n0}C_{b}^{b0}f^{b}-\left(\delta \omega_{ie}^{n0}\times V^{n0}+\omega_{ie}^{n0}\times \delta V^{n0}\right)\\ & \approx -\phi^{i}\times C_{b0}^{n0}C_{b}^{b0}f^{b}+C_{b0}^{n0}C_{b}^{b0}\left(\nabla^{b}+w_{a}^{b}\right)-\omega_{ie}^{n0}\times \delta V^{n0}\\ & =\left[\left(C_{b0}^{n0}C_{b}^{b0}f^{b}\right)\times \right]\phi^{i}-\omega_{ie}^{n0}\times \delta V^{n0}+C_{b0}^{n0}C_{b}^{b0}\left(\nabla^{b}+w_{a}^{b}\right) \end{array}$$

where $\delta {\dot{V}}^{n0}$ represents the error form of the vector ${\dot{V}}^{n0}$. $\nabla^{b}$ represents the zero bias of the accelerometer. $w_{a}^{b}$ represents the measurement noise of the accelerometer. $\delta \omega_{ie}^{n0}$ represents the error form of angular velocity $\omega_{ie}^{n0}$.

The motion displacement vector $d^{n0}$ of the carrier can be obtained by integrating the velocity vector $V^{n0}$. The displacement vector in the n0 system is defined as the following:

(18) $$d^{n0}=\int_{0}^{t}V^{n0}dt$$

In practical application, the displacement vector can be calculated with the following expression:

(19) $${\tilde{d}}^{n0}=\int_{0}^{t}{\tilde{V}}^{n0}dt$$

The derivatives of Equation (18) and Equation (19) are expressed as the following:

(20) $${\dot{d}}^{n0}={\left(\int_{0}^{t}V^{n0}dt\right)}^{\prime }=V^{n0}$$

(21) $${\dot{\tilde{d}}}^{n0}={\left(\int_{0}^{t}{\tilde{V}}^{n0}dt\right)}^{\prime }={\tilde{V}}^{n0}$$

Subtracting Equation (21) from Equation (20) yields

(22) $$\delta {\dot{d}}^{n0}={\dot{\tilde{d}}}^{n0}-{\dot{d}}^{n0}={\tilde{V}}^{n0}-V^{n0}=\delta V^{n0}$$

The state vectors of the inertial frame based on forward–forward backtracking fine alignment are defined as the following:

(23) $$X={\left[\begin{array}{ccc} \phi^{i} & \delta V^{n0} & \begin{array}{ccc} \delta d^{n0} & \varepsilon^{b} & \nabla^{b} \end{array} \end{array}\right]}^{T}$$

In summary, the state equation of the fine alignment filtering model can be obtained as the following:

(24) $$\dot{X}=FX+W$$

where the state transfer matrix F and the system noise array W are expressed as

(25) $$F=\left[\begin{array}{ccccc} 0 & 0 & 0 & -C_{b0}^{n0}C_{b}^{b0} & 0\\ \left(C_{b0}^{n0}C_{b}^{b0}f^{b}\right)\times & \omega_{ie}^{n0}\times & 0 & 0 & C_{b0}^{n0}C_{b}^{b0}\\ 0 & I & 0 & 0 & 0\\ 0 & 0 & 0 & 0 & 0\\ 0 & 0 & 0 & 0 & 0 \end{array}\right]$$

(26) $$W={\left[\begin{array}{ccccc} -C_{b0}^{n0}C_{b}^{b0}w_{g}^{b} & C_{b0}^{n0}C_{b}^{b0}w_{a}^{b} & 0 & 0 & 0 \end{array}\right]}^{T}$$

### 2.2. Measurement Equation of Backtracking Alignment

The observation equation of the fine alignment filtering model is given by

(27) $$Z=\delta d^{n0}=d_{GNSS}^{n0}-d^{n0}=\left[\begin{array}{ccccc} 0 & 0 & I & 0 & 0 \end{array}\right]X+v$$

where v denotes the measurement noise and $d_{GNSS}^{n0}$ denotes the displacement vector in the n0-frame computed from the GNSS’s position.

For the displacement vector $d_{GNSS}^{n0}$ in Equation (27), it is necessary to derive the expression for the carrier displacement vector in the n0-frame, since the GNSS can only obtain position information in the n-frame.

**Figure 2:**
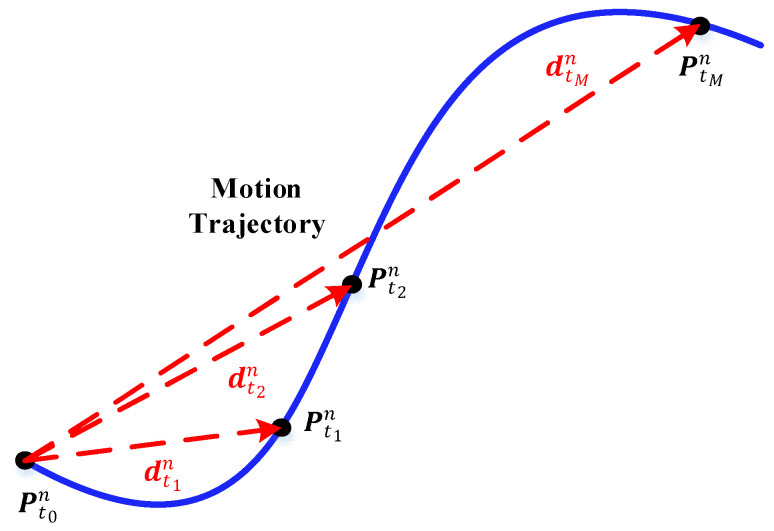
Schematic diagram of the displacement vector solution principle for the GNSS.

A schematic diagram of the displacement vector solution principle for the GNSS is given in Figure 2. Using the displacement vector relationship diagram given in Figure 2, the displacement vector of the underwater vehicle in the n0-frame can be calculated as

(28) $$\begin{array}{c} d_{GNSS}^{n0}\left(t_{1}\right)=C_{n\left(t_{1}\right)}^{n0}d_{t_{1}}^{n}=C_{n\left(t_{1}\right)}^{n0}\left(P_{t_{1}}^{n}-P_{t_{0}}^{n}\right)\\ d_{GNSS}^{n0}\left(t_{2}\right)=C_{n\left(t_{2}\right)}^{n0}d_{t_{2}}^{n}=C_{n\left(t_{2}\right)}^{n0}\left(P_{t_{2}}^{n}-P_{t_{0}}^{n}\right)\\ \cdots \\ d_{GNSS}^{n0}\left(t_{M}\right)=C_{n\left(t_{M}\right)}^{n0}d_{t_{M}}^{n}=C_{n\left(t_{M}\right)}^{n0}\left(P_{t_{M}}^{n}-P_{t_{0}}^{n}\right) \end{array}$$

where $d_{t_{M}}^{n}$ represents the projection of the carrier displacement in the n-frame at $t_{M}$. $P_{t_{M}}^{n}$ represents the position of the carrier measured using the GNSS at $t_{M}$.

## 3. Simulation Test

In the previous section, a detailed explanation of the derivation process of the forward–forward backtracking fine alignment algorithm based on the displacement measurements in an inertial frame was given. A simulation test was then utilized to verify the effectiveness of the designed method.

The parameter settings of each sensor in the fine alignment simulation test were as shown in Table 1.

The starting position was set as the following: ${\left[\begin{array}{ccc} 118.786365\;{}^{\circ}E & 32.057313\;{}^{\circ}N & 0 \end{array}\right]}^{T}$. The initial speed was ${\left[\begin{array}{ccc} 0 & 0 & 0 \end{array}\right]}^{T}m/s$. The initial motion direction of the UGV was west. The trajectories of the UGV in the simulation test are shown in Table 2.

**Figure 3:**
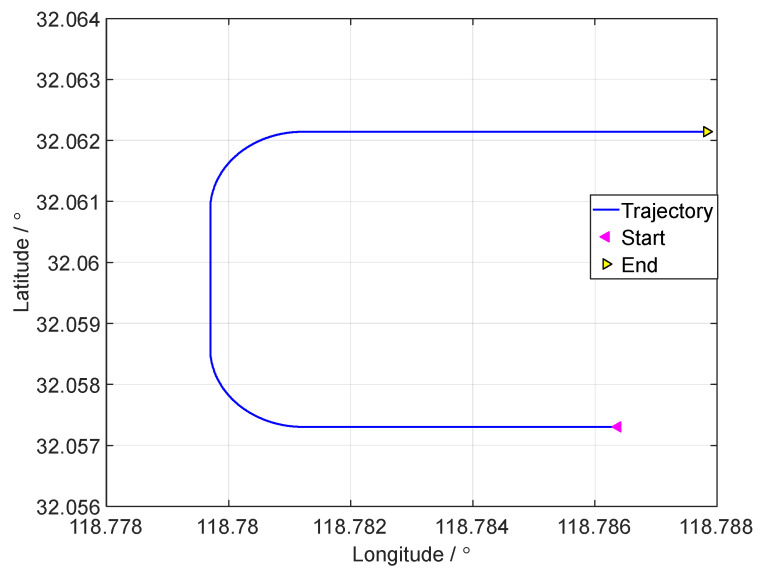
The motion trajectory curve of the simulation test.

**Figure 4:**
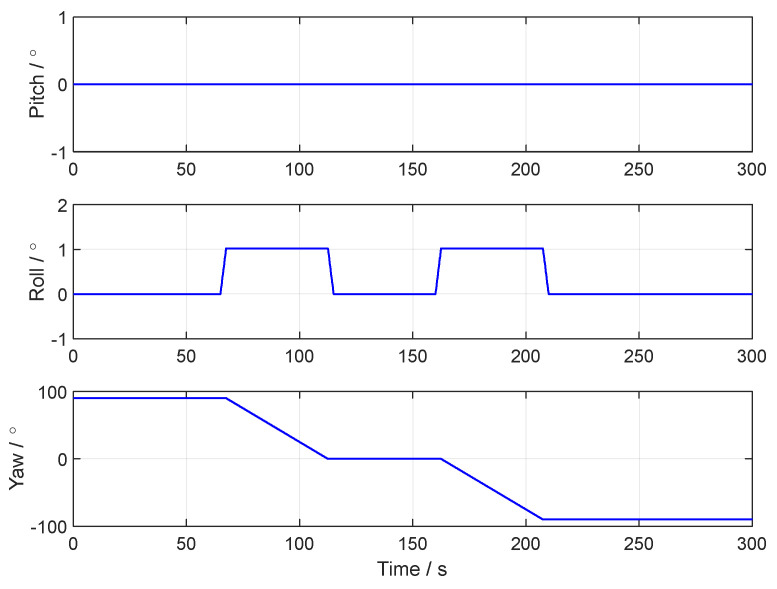
The attitude curve of the UGV in the simulation test.

**Figure 5:**
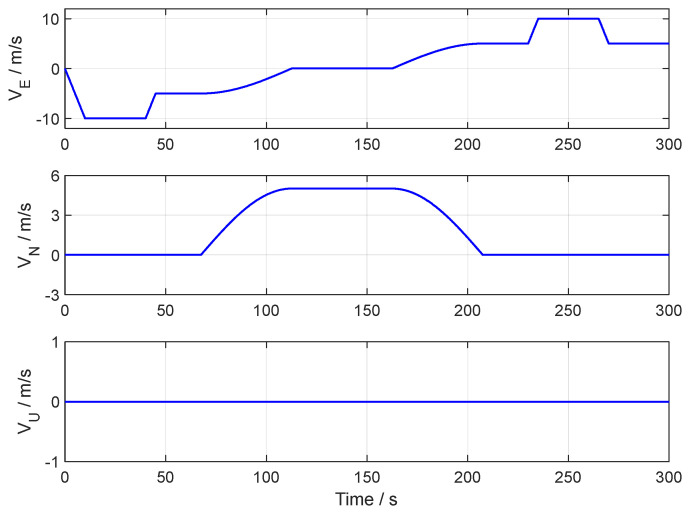
The velocity curve of the UGV in the simulation test.

Figure 3, Figure 4 and Figure 5, respectively, show the motion trajectory and parameters of the UGV in the simulation test.

**Figure 6:**
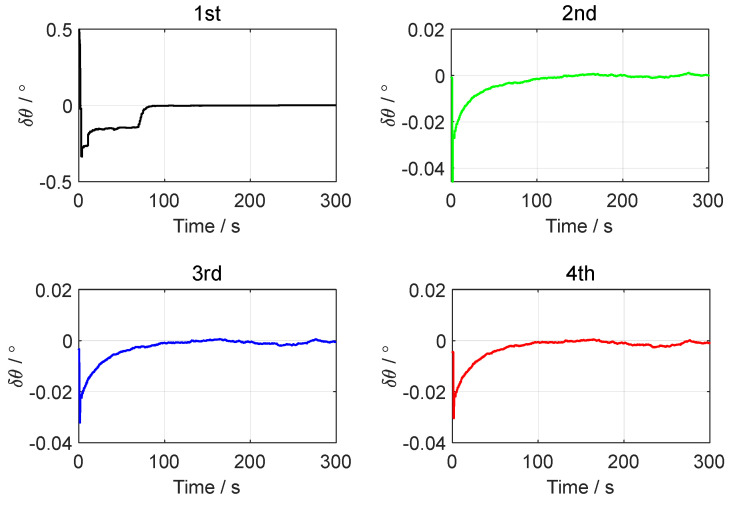
The pitch angle errors in the simulation test.

**Figure 7:**
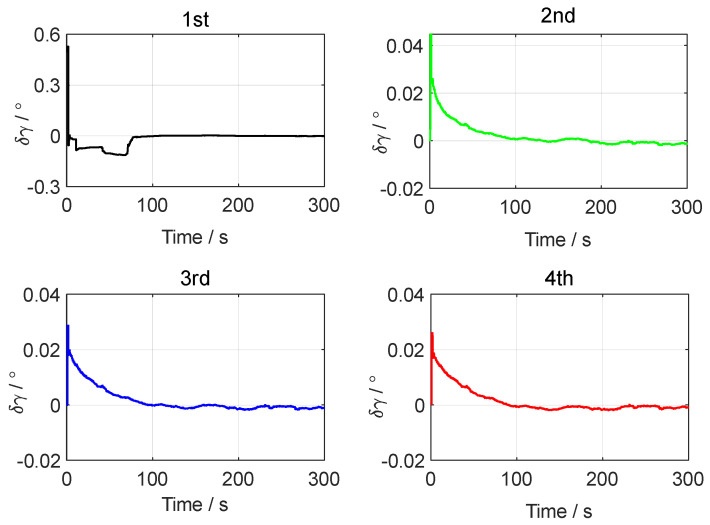
The roll angle errors in the simulation test.

**Figure 8:**
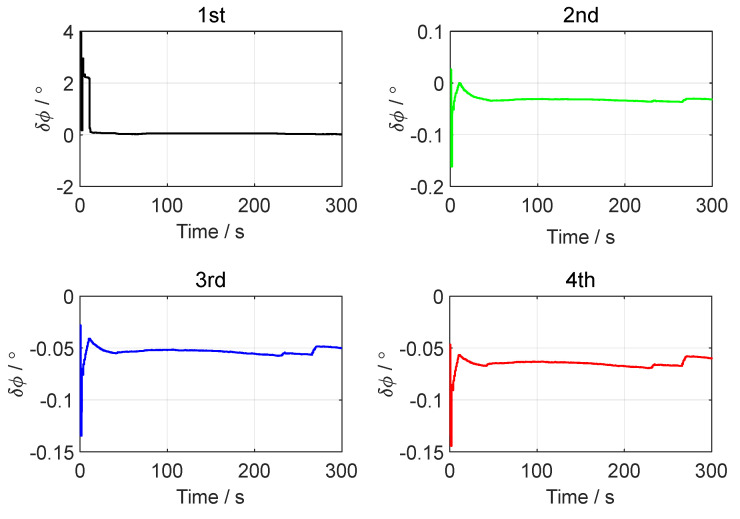
The yaw angle errors in the simulation test.

In the simulation test of in-motion fine alignment, the initial attitude error was set to ${\left[\begin{array}{ccc} 0.1^{{}^{\circ}} & 0.1^{{}^{\circ}} & 1^{{}^{\circ}} \end{array}\right]}^{T}$. The initial velocity error was set to ${\left[\begin{array}{ccc} 0.1 & 0.1 & 0.1 \end{array}\right]}^{T}m/s$, and the initial position error was set to ${\left[\begin{array}{ccc} 10 & 10 & 10 \end{array}\right]}^{T}m$. The forward–forward backtracking alignment was performed four times. The whole fine alignment process lasted 300 s. Figure 6, Figure 7 and Figure 8 show the attitude errors for the four forward–forward backtracking alignments on the moving base.

From Figure 6 and Figure 7, it can be seen that the horizontal attitude angle error converged to within 0.02° after the first backtracking alignment. The heading angle error curve in Figure 8 shows that the yaw angle error reached within 0.1° after two forward–forward backtracking fine alignments. To verify the advantage of the forward–forward backtracking fine alignment method in terms of alignment speed, the MATLAB profile command was used to determine the running time of the backtracking fine alignment algorithm. The results showed that the running time of each forward–forward backtracking fine alignment was less than 4 s. Including the coarse alignment time of 300 s used for storing data, the in-motion initial alignment process was completed within 320 s. This greatly improved the convergence speed of in-motion initial alignment.

## 4. Vehicle Test

**Figure 9:**
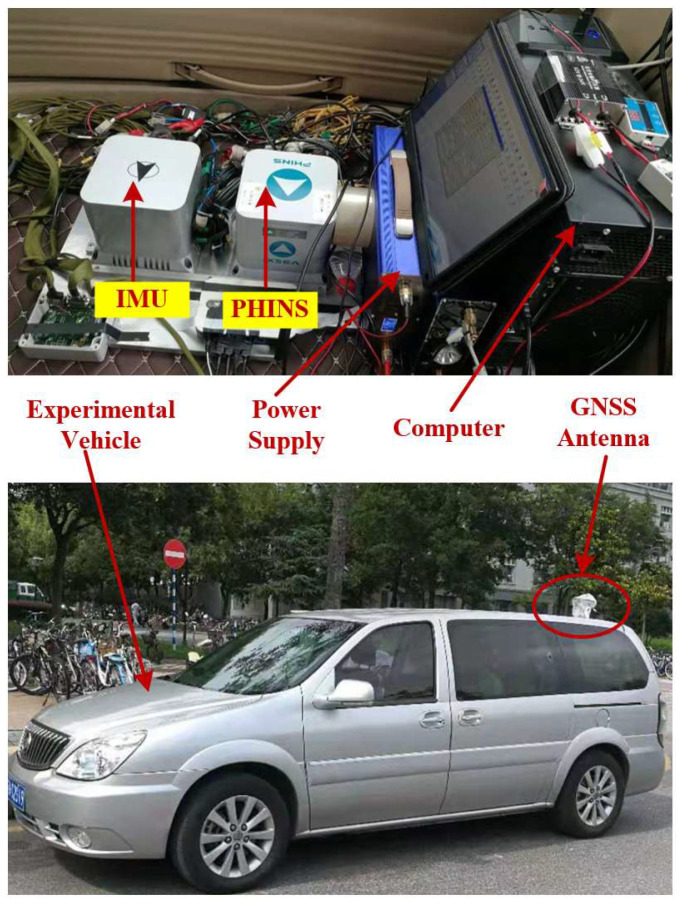
The equipment in the vehicle test.

The results of the simulation test confirmed the effectiveness of the backtracking alignment algorithm, but whether the method could be applied to real-world environments needed to be verified. This section discusses the vehicle test utilized to verify the feasibility and reliability of the backtracking alignment algorithm in a real environment. The physical diagram of the vehicle and equipment used for the test are shown in Figure 9. Figure 9 shows the equipment used in the vehicle test, including an IMU, an attitude reference (PHINS), a power supply, a navigation computer, and a GNSS antenna. The GNSS antenna was mounted on top of the vehicle. The IMU included a three-axis gyroscope and a three-axis accelerometer, whose specific parameters are shown in Table 3. The PHINS was an INS/GPS integrated navigation system produced by the iXblue company in Paris, France. In the vehicle test, the attitude output from the PHINS and GPS integrated navigation system was used as the reference attitude for the alignment algorithm. The IMU and PHINS were mounted on a board, and the installation error between them was calibrated, compensating for the three-axis turntable in advance.

**Figure 10:**
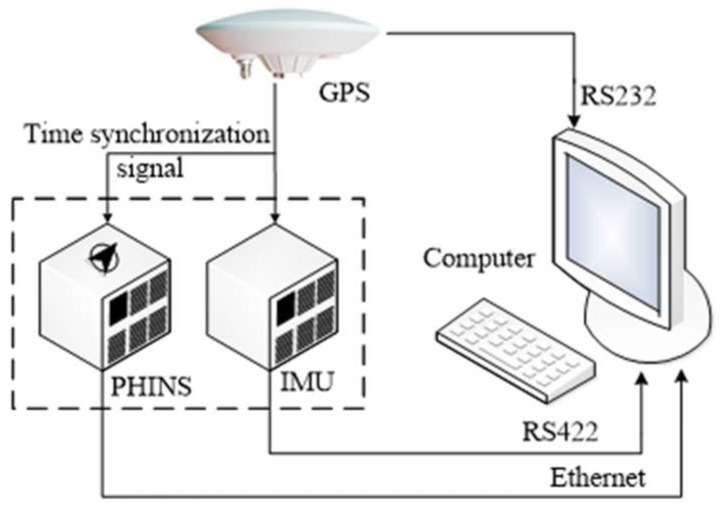
The process of the vehicle test.

The process of the vehicle test is shown in Figure 10. As shown in Figure 10, the GPS signal was used as the time synchronization signal of the IMU and PHINS. Meanwhile, the GPS position output was sent to the PHINS and the navigation computer through the RS232 port. The raw data from the IMU were sent to the navigation computer via the RS422 port. The reference attitude, reference velocity, and reference position information from the PHINS-integrated navigation system were sent to the navigation computer via the Ethernet. The navigation computer executed the backtracking alignment algorithm proposed in this paper using the raw data of the IMU and the position information of the GPS. The calculated attitude results were compared with the reference attitude of the PHINS to obtain the attitude error of the alignment algorithm. Finally, all the device data and alignment results were saved in the navigation computer.

**Figure 11:**
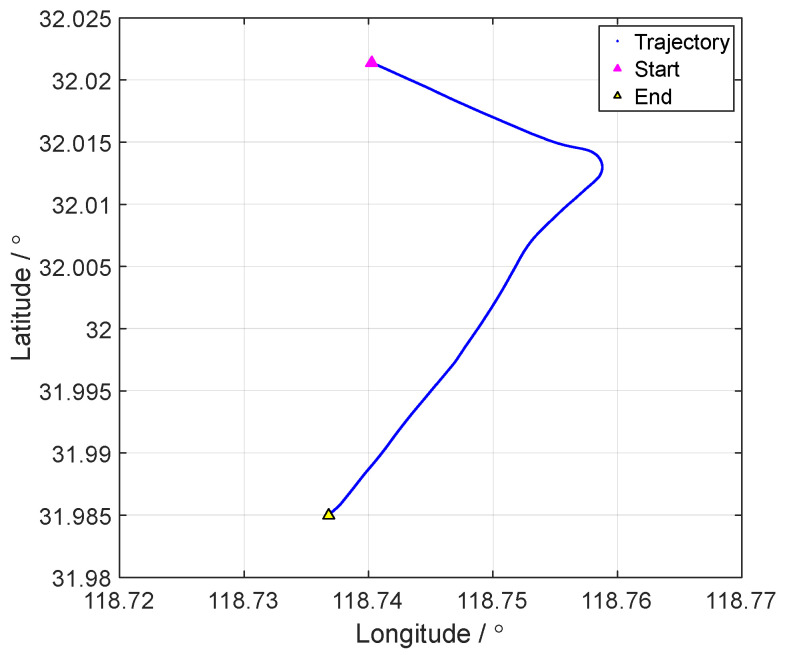
The motion trajectory curve of the vehicle test.

**Figure 12:**
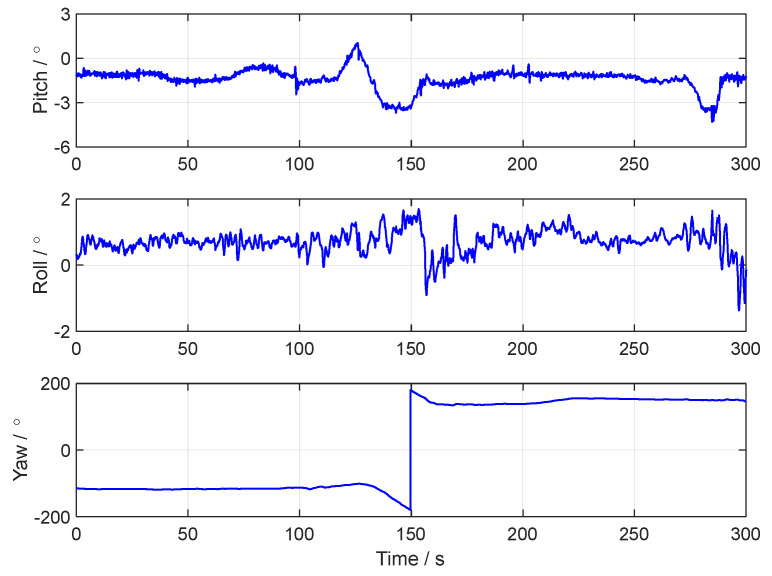
The attitude curves of the vehicle test.

**Figure 13:**
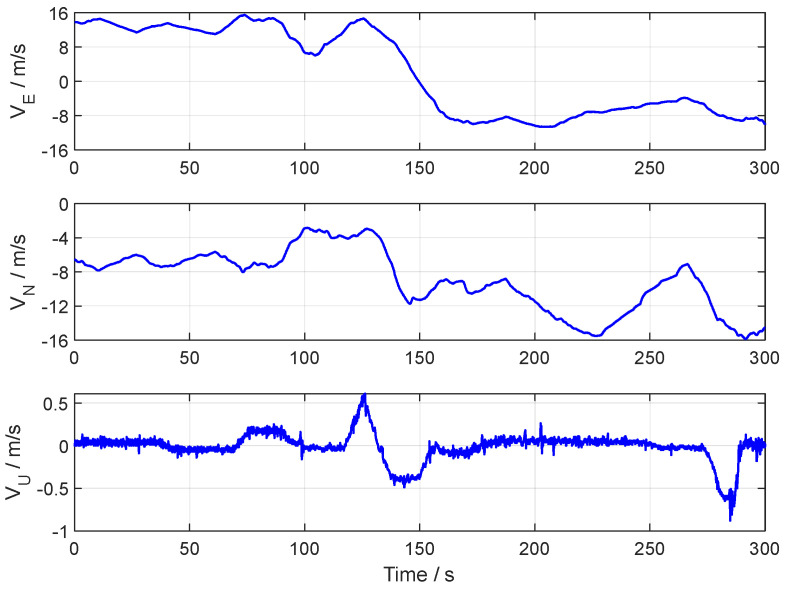
The velocity curves of the vehicle test.

The vehicle tests were conducted on elevated roads in Nanjing City, Jiangsu Province. The running trajectory in the vehicle test is shown in Figure 11. The reference attitude and speed in the vehicle test are shown in Figure 12 and Figure 13, respectively.

**Figure 14:**
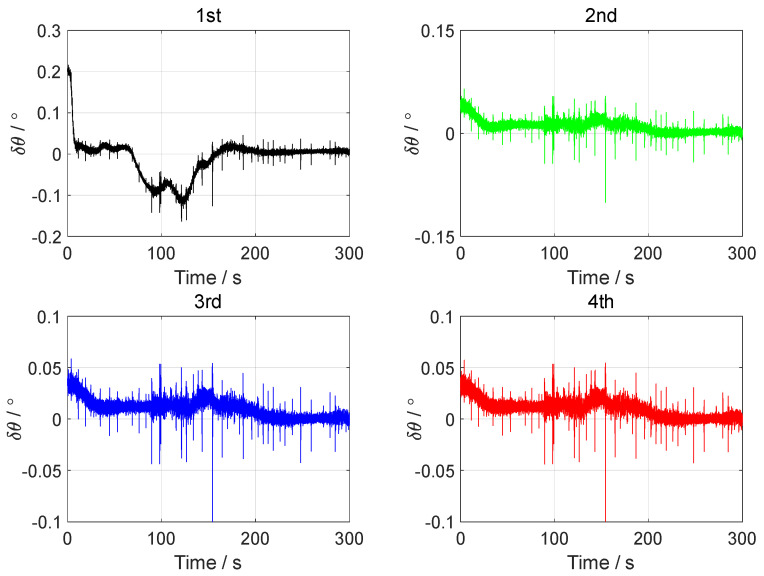
The pitch angle errors in the vehicle test.

**Figure 15:**
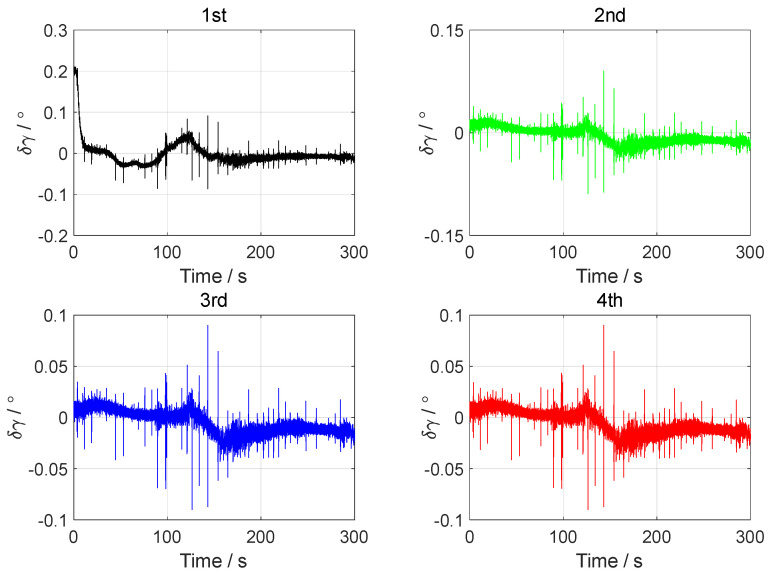
The roll angle errors in the vehicle test.

**Figure 16:**
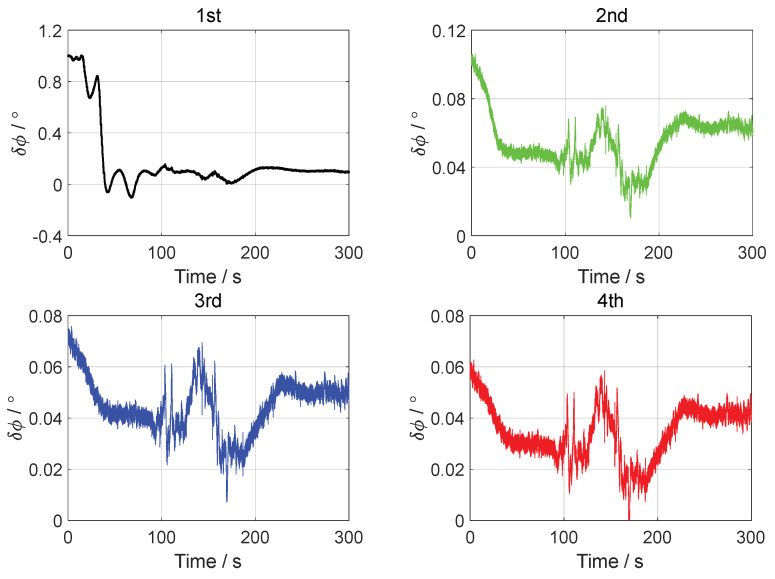
The yaw angle errors in the vehicle test.

In the vehicle test, the initial attitude error was set to ${\left[\begin{array}{ccc} 0.1^{{}^{\circ}} & 0.1^{{}^{\circ}} & 1^{{}^{\circ}} \end{array}\right]}^{T}$. The initial velocity error was set to ${\left[\begin{array}{ccc} 0.1 & 0.1 & 0.1 \end{array}\right]}^{T}m/s$, and the initial position error was set to ${\left[\begin{array}{ccc} 10 & 10 & 10 \end{array}\right]}^{T}m$. The forward–forward backtracking alignment was performed four times. The whole fine alignment process lasted 300 s. The attitude error curves of the backtracking fine alignment in the vehicle test are shown in Figure 14, Figure 15 and Figure 16.

From the error curves of the horizontal angle in Figure 14 and Figure 15, it can be seen that the pitch angle error converged to about ±0.05° and the roll angle error converged to about ±0.04° after two backtracking alignments. The error curves of the yaw angle in Figure 16 show that the yaw angle error of the position-assisted backtracking fine alignment algorithm remained within 0.08° after two backtracking alignments.

For clearer and more specific statistics on the performance of the backtracking fine alignment algorithm in the vehicle test, see Table 4, which lists the attitude error statistics for the four backtracking fine alignments in the vehicle test in the 200–300 s interval. In Table 4, MN denotes the mean of the attitude angle error. STD denotes the standard deviation of the attitude angle error. RMS denotes the root mean square of the attitude angle error.

From Table 4, it can be seen that the yaw angle accuracy of the backtracking fine alignment algorithm was optimized every time. After the second backtracking alignment, the yaw angle error remained within 0.07°, indicating that the fine alignment performance met the requirements. The results of the vehicle test, i.e., the attitude error in the figure and the error statistics table, proved the feasibility and reliability of the backtracking fine alignment algorithm proposed in this paper.

## 5. Conclusions

In existing backtracking alignment methods, most approaches are based on forward–backward backtracking alignment, which requires storing full raw IMU data and has high memory demands. A few forward–forward backtracking methods use a Doppler velocity log or an odometer as external measurement information, but none utilize GPS positioning for backtracking alignment. In this paper, a GNSS displacement-assisted forward–forward backtracking fine alignment algorithm is proposed to improve the convergence speed of the initial alignment process. First, a forward–forward backtracking fine alignment model was constructed in the initial navigation frame. The displacement vector in the initial navigation frame solved using the GNSS position was taken as the observation of the fine alignment. Simulation and vehicle tests were carried out to verify the performance of the proposed fine alignment algorithm. The experimental results showed that this algorithm could effectively improve the accuracy and convergence speed of the fine alignment process while storing only a small amount of raw data.

## Figures and Tables

**Table 1:** The settings of the sensor parameters in the simulation test.

Sensor	Parameters	Value
IMU	Gyroscope biases	0.02 °/h
	Gyroscope random noise	0.005 °/√h
	Accelerometer biases	500 μg
	Accelerometer random noise	50 μg/√Hz
	Output frequency	200 Hz
GPS	Positioning error	10 m
	Speed error	0.5 m/s
	Output frequency	1 Hz

**Table 2:** The settings of the motion state in the simulation test.

Order	Time (s)	Motion State	Order	Time (s)	Motion State
1	0–10	$\mathrm{Accelerated}\;\mathrm{motion},\;a=1\;m^{2}/s$	7	160–205	Turning right, w = 2 °/s
2	10–40	Uniform motion	8	205–225	Uniform motion
3	40–45	$\mathrm{Decelerated}\;\mathrm{motion},\;a=1\;m^{2}/s$	9	225–230	$\mathrm{Accelerated}\;\mathrm{motion},\;a=1\;m^{2}/s$
4	45–65	Uniform motion	10	230–255	Uniform motion
5	65–110	Turning right, w = 2 °/s	11	255–260	$\mathrm{Decelerated}\;\mathrm{motion},\;a=1\;m^{2}/s$
6	110–160	Uniform motion	12	260–300	Uniform motion

**Table 3:** The parameters of the IMU in the vehicle test.

Sensor	Parameters	Value
Gyroscope	Constant biases	<0.02 °/h (1σ)
	Random noise	<0.005 °/√h
	Measurement range	±300 °/s
	Output frequency	200 Hz
Accelerometer	Constant biases	<5 × $10^{-5}\;g\left(1\sigma \right)$
	Random noise	<5 × $10^{-5}\;g\left(1\sigma \right)$
	Measurement range	±20 g
	Output frequency	200 Hz

**Table 4:** The attitude error statistics of the fine alignment in the vehicle test.

Order	Error	Pitch	Roll	Yaw
1st	MN (°)	0.0055	−0.0087	0.1114
	STD (°)	0.0035	0.0038	0.0112
	RMS (°)	0.0065	0.0095	0.1120
2nd	MN (°)	0.0019	−0.0116	0.0636
	STD (°)	0.0034	0.0039	0.0036
	RMS (°)	0.0039	0.0123	0.0637
3rd	MN (°)	0.0012	−0.0120	0.0489
	STD (°)	0.0035	0.0039	0.0045
	RMS (°)	0.0037	0.0127	0.0491
4th	MN (°)	0.0010	−0.0123	0.0400
	STD (°)	0.0035	0.0040	0.0048
	RMS (°)	0.0037	0.0129	0.0403

## Data Availability

The data presented in this study are available on request from the corresponding author. The data are not publicly available to protect the privacy of the subjects involved in this study.
